# Memoir on Tumors of the Gums, Known as Epulis

**Published:** 1858-10

**Authors:** 


### AMERICAN JOURNAL

OF

### DENTAL SCIENCE.

Vol. VIII. NEW SERIES—OCTOBER, 1858. No 4.

### ORIGINAL COMMUNICATIONS.

### ARTICLE I.

*Memoir on Tumors of the Gums, known as Epulis.* By Dr. Saurel. (Translated from the French for the American Journal of Dental Science.)

[Continued from the April Number.]

Doctor Rigal published in 1827, in the *Revue Medicale*, under the title of "epulis from caries of the maxillary bones," two cases of tumors of the gums, presenting in a high degree the characters of carcinomatous epulis. I shall state them as briefly as possible.

Case XV. *Carcinomatous Epulis of the lower jaw. Ligature. Recurrence. Second ligature imperfect. Increase of the Tumor. Third ligature. Death.* By M. Rigal.

Jeanne Marie Taillefer, eighty-three years old, towards the end of 1819, experienced frequent pains in the lower jaw. She had never suffered from disease of the teeth, and had lost hers by the mere advance of old age. Soon she perceived arising from the lower jaw a tumor, which increasing slowly at first, acquired ultimately a rapid development, and so interfered with deglutition as to hinder the old woman from sustaining life.

M. Rigal, summoned to her on November 19th, 1820, made the following observations: skin sallow, (terreuse;) emaciation extreme; pulse small, weak, infrequent. On opening the mouth he perceived a large tumor which entirely filled it.

"This tumor, of a livid, hard, resisting appearance, sprung from the left ramus of the lower jaw, covering the whole of the dental arch as far as the incisors. Besprinkled here and there with irregular phlyctænæ, it presented two lobes, each of which was as large as a turkey's egg. The patient could not speak, was on the point of suffocation twenty times a day, and was only able to ingest into the stomach broths taken at remote intervals and very small quantity.

"Regularly every evening, and sometimes during the day, this mass became the seat of acute and lancinating pains. It might, however, be pressed without giving rise to great suffering, but its resistance did not permit it to be lifted or in anywise displaced."

M. Rigal, thinking the mass to be pedicled, decided to tie it, an operation which he successfully accomplished. Three days after, the tumor was black, sphacelated and emitted an insupportable odor. It was sufficient to grasp it with the fingers to detach and remove it. It was withered and had lost one-fourth its original volume. "After its fall," says the author, "I saw that the pedicle sprung from the site of the second molar tooth and, as I had supposed, the carious lower jaw was the true cause of this enormous epulis." Under this apprehension, he was provided with cauterizing irons to destroy the bony lesion, which did not occupy more than one-third the height of the left ramus of the jaw; but the weakness of the patient rendered him apprehensive that she could not tolerate this heroic remedy.

She recovered pretty well, but she did not profit by this amelioration to attempt a cure. The epulis reappeared and its development was extremely active. Eight months afterwards, another physician applied a ligature which, not reaching the pedicle of the tumor, caused the fall of only a part of the excrescence. The progress of the malady was thereby singularly aggravated. "The pains became acute, profound, continued. The epulis increased at a frightful rate of progression. M. Rigal, summoned again fourteen months after his first visit, conducted matters as before and with the same success, so far as concerned the fall of the tumor, which presented the same appearance. But this time the whole left ramus of the lower jaw was affected; its swelling was as large as a pullet's egg: the pedicle had acquired a proportionate dimension, and there remained no hope of a radical cure. Age, pain and the exhaustion resulting from the defect of nutrition, soon carried the patient to the tomb."

The second case of M. Rigal closely resembles the first, except that the tumor was less voluminous and that the cure was obtained by combining excision with cauterization.

Case XVI. *Carcinomatous Epulis of the upper jaw, cured by excision, followed by cauterization.* By M. Rigal.

Joseph Molinier, forty-two years of age, had on the right side of the upper jaw an epulis which issued from the place of the first two molars. Three years ago he had these extracted on account of caries followed by very intense pains, which, however, had never produced parulis. He had remarked the excrescence only for twenty-five or twenty-six months. Indolent and growing slowly at first, it soon sensibly increased in size and occasioned pain.

The tumor was smooth and polished, wine-red in color and irregularly embossed, and had the form and size of a pullet's egg. Thrown outwards by the lower jaw which bit its internal border, it presented at this point an excoriation bathed with a sanious and bloody humor. Its hardness was decided, and at night especially it became the seat of acute and lancinating pains. The pedicle was concealed by the folds which formed the epulis upon the sides of the alveolus; for the rest, the gums were firm, not swollen and in a word in the soundest possible condition.

The author, not doubting that the bone was the seat of the disease, decided to remove the tumor and destroy the caries by the actual cautery. This was carried out with some difficulty. On passing the finger over the wound resulting from the removal of the tumor, some inequalities produced by the caries were felt. The disease did not appear even to involve the entire thickness of the alveolar border. The hemorrhage, which was abundant, yielded to cauterization. The patient left for his own country. The necrosis was removed by the unaided efforts of nature, and one year afterwards, the cure was complete.

On examining this epulis, it was found to be made up of a dense, cartilaginous and bony tissue at several points, especially about the middle; the places which did not present these degenerations offered a striking resemblance to lardaceous cancerous tissue.

M. Rigal follows the recital of these two cases by remarks in which he acknowledges that the term *caries*, which he has used to designate the alteration of the maxillæ among his patients, is improper, and that the pathological condition in question has the greatest analogy with osteosarcoma in general, and the sclerosarcoma of Mernget in particular.

The presence of newly formed bony tissue in the tumors of the gums is an important fact which seems to indicate that epulis, wherever we encounter it, proceeds directly from the maxillary bones. This will be observed in the following case, occurring in the practice of M. H. Larrey.

It had already been mentioned by M. Begin, in the article heretofore quoted from the *Dictionnaire de Médecine et de chirurgie practiques.*

CASE XVII. *Carcinomatous Tumor of the gums, seated at the concavity of the lower jaw. Excision and cauterization. Recurrence. Second cauterization. Doubtful cure.*

Robendo, twenty-eight years of age, of a strong constitution, was attacked eight months ago with a tumor which commenced by a little pimple seated on the concavity of the lower jaw, on the portion of the gums between the two left incisors. It has progressively increased without causing pain.

May 1st, 1840, this tumor is as large as a nut. It is situated behind the lower alveolar arch, to which it adheres in front, rising almost to the level of the incisors, and terminating behind near the frænum of the tongue. In front the tumor has a prolongation between the two incisors, shaped like a mushroom, and projecting far enough to displace the lower lip. These two teeth are displaced and loosened, although sound. The tumor, irregular in form, embossed, violet in color, has several ulcerated points, some bloody and others grayish and livid. It is soft and spongy on the surface, firmer and more resisting at the centre; it bleeds easily and its vascularity is evident to the eye. The patient's breath is fetid; his mouth is always full of a sanious humor, he experiences difficulty in pronunciation and mastication.

This tumor has been formed without appreciable cause, general or local. It appears to be developed in the tissue of the gum, which alone is attacked. It was extirpated by a cutting instrument of peculiar form, avoiding the bone; the wound being cauterized with red-hot iron.

The tumor, examined a little after the operation, was discolored and withered, resembling a lymphatic ganglion. It resisted the edge of the bistoury. Its section showed a tissue soft on the surface, compact in the subjacent layers, fibrous at greater depth and very hard at the centre. There it had a nucleus of a bony appearance, with radiating striæ, which communicated to the finger a sensation of roughness and when struck with the probe emitted a clear sound like that of a calcareous concretion.

The results of the operation seemed at first very favorable, but, at the end of a few days, some vegetations made their appearance about the primitive site of the tumor. A new cauterization with red-hot iron destroyed them, and the cure was established, save the probable chances of recurrence, says the reporter of the case.

I shall complete the account of the characters of carcinomatous epulis by quoting a note of M. Charles Robin, published in the *Comptes rendus de la Société de Biologie.*

CASE XVIII. *Micrographical examination of an Epulis of the lower jaw.* By M. CH. ROBIN.

"M. Robin presented a tumor of the size of a small nut, which had been sent him by M. Dionis, *interne* of the hospitals. This tumor which compelled the amputation of a portion of the lower jaw, because it was considered cancerous, was in reality destitute of the characteristic juice of this degeneration. On examining a portion of the tissue of the surface, Messrs. Dionis and Robin found there lamellæ with multiple nuclei, such as are found in the normal state in the marrow of bones. They diagnosticated that the disease originated in the bony tissue of the maxilla and not in the periosteum, as was at first supposed. A section of the bone showed that the tumor rose from the bone and had invaded a fourth of its tissue. There were no cancerous elements. The morbid tissue was exclusively formed of—first, lamellæ with very numerous multiple nuclei; second, fibroplastic elements, (nuclei and fusiform fibres;) third, cellular tissue less abundant than the previously named elements; and fourth, capillary vessels and molecular granules.

This last fact, interesting from the light it throws upon the intimate structure of the tumors we are considering, can be but of moderate utility in a clinical point of view, for nothing is said of the circumstances under which the epulis was developed, nor of the physical properties of the tumor and the phenomena to which it gave rise. The sole point of interest is the fact that the tumor rose from the bone, a portion of the thickness of which it invaded.

By comparing the facts we have cited and bringing to light their salient points, we shall be able to establish the clinical characters of *carcinomatous epulis.*

Like the preceding, these tumors may occur without known cause, preceded or not by dental caries. Acute, severe, lancinating pains, continuous or recurring at intervals, especially at night, almost always accompany their appearance and development. Not particularly remarkable at their commencement, they raise the gums over a greater or less extent; their surface is smooth without asperities. Covered by the mucous membrane of the gums, which they at first resemble in color, later, they assume a characteristic violet tint. Their increase, slow at first, soon becomes rapid and they may acquire enormous dimensions, even to the extent of filling the buccal cavity and producing hideous deformity. Irregular fissures and ulcerations form on their surface and emit a sanious fetid pus. They bleed readily, but when pressed between the fingers they cannot be diminished; they are hard, resisting and absolutely incompressible.

At the same time that they are thus developed in the buccal cavity, they determine in the bones in which they are implanted, more and more profound alterations. The bony tissue is destroyed and absorbed, some of its laminæ mortify, so that it may present the appearance of caries and necrosis. Consecutively to these alterations of the bones, abscesses are formed, new fungosities appear and the disease extends more and more, both superficially and in depth. All causes of irritation only increase the activity of their development. Ligature and ablation are inevitably followed by recurrence, unless the roots of the disease have been destroyed by operating upon the bone itself. Marasmus and death may supervene in consequence of these tumors when they have not been properly treated in time.

The anatomical examination of these tumors shows that they are composed of a somewhat lardaceous and cartilaginous tissue, enclosing osseous nuclei and taking root in the bone, which is attacked and partly destroyed.

III. Pathology. The examination just completed was designed, by the aid of clinical facts, to establish the existence of different varieties of tumors of the gums and to determine the characters peculiar to each. It remains, in order to complete this work, to profit by the notions already acquired for the purpose of tracing the pathology of epulis and establishing rules for the treatment of the disease.

Let us begin by recalling our definition. "I give the name of epulis (from επε, upon and ο'υλον, gum) to all the fungous, vascular, hard or carcinomatous tumors, which springing from the gums, the alveolar dental periosteum or the alveolar arches, are developed in the interior of the mouth."

The *causes* of epulis are not well determined. More frequently, especially in the graver cases, this disease appears without appreciable general or local cause. If we believe Léveillé, scrofulous patients are most liable to it, and it may also originate in venereal or other affections which have become constitutional. Dental caries is a cause which figures in a certain number of cases ; at other times, the epulis has been preceded by a fluxion on the gums, followed or not by abscess or parulis. Caries and necrosis of the alveoli have also been designated as cancer. So also have repeated irritations of the gums, the use of too hot food, &c. Contusions and wounds of the gums may also cause epulis ; we have seen a tumor of this sort succeed the lesion occasioned by fragments of nut shell. Finally, fractures of the jaw may cause it. Job à Meekren reports that a tall, large man, enjoying good health, having fractured his lower jaw and lost several teeth by a fall, got a fleshy excrescence as big as a man's fist, which greatly disfigured him.

*Age* does not appear to exert great influence upon the formation of epulis; yet this disease seems to me less common in infancy and youth than adult and advanced age. The same cannot be said of *sex*. In twenty-two cases in which the sex has been noted, I find fifteen women and only seven men.

These tumors have their *seat* oftener on the lower than on the upper jaw. In twenty-one cases in which their position is indicated, I find thirteen on the lower jaw, seven on the upper and one on both. They may be developed at all points of the alveolar arch, almost indifferently; nevertheless, it has appeared to me that carcinomatous epulis has a preference for the molars. It originates between the teeth, or upon the anterior or posterior faces of the gum, the latter being less frequent.

The *volume* of these tumors is very variable. At the beginning, or in the early stages of the disease, they are necessarily small; later, they may attain the size of a walnut, a horse-chesnut, an egg or even a greater volume. In Job à Meekren's case, already cited, the tumor was so large that the opening of the mouth was not sufficient for its extraction till after its division. In one of the cases reported by M. Rigal, the epulis was double, each portion equalling in size a turkey's egg.

The *form* of these tumors is not less variable. Usually, they are rounded and salient; sometimes they are flattened like a mushroom or present several distinct lobes. Frequently they are marked with furrows of greater or less depth, occasioned by the teeth of the opposite jaw. The pressure of the neighboring organs, such as the cheeks, the lips and the tongue, also influences their form.

A great number of these tumors have a pedicle more or less narrow and elongated; others, on the contrary, are sessile or even implanted by a very large base, since they have been seen to occupy all or nearly all the length of the alveolar arch.

The pediculated epulides are generally *mobile*, whatever may be their nature; it may be, however, that their great volume resists displacement, even when they have a pedicle. Sessile tumors and those with a large base are almost always immovable; this is always the case with those which are carcinomatous.

The *color* of these tumors is not always the same. Some present the natural line of the gums and of the mucous membrane of the mouth, that is, a pale rosy tint; others are of a lively red; many are of a deep red bordering on brown; finally, there are some which present an obscure violet. This coloration is not unimportant in reference to diagnosis and prognosis. Attentive study has shown me that the simple fibrous and fibro-plastic tumors generally retain the normal color of the gums or assume a bright red; that the reddish tint belongs to the fungous and erectile epulides, and that the violet coloration almost always characterizes the carcinomatous tumors.

The *consistence* of these tumors, up to a certain point, corresponds with their nature. Some, soft and compressible, are easily torn by the fingers and by instruments; these are fungous epulides. Others more resisting, but diminishing in volume under the pressure of the explorer's finger, communicate a sensation of trembling or arterial pulsation; these are the erectile or vascular. Others are hard, incompressible; this character belongs to fibrous, fibro-plastic and bony tumors. Finally, in some cases the tumor appears soft, fungous and compressible on the surface, while a little stronger pressure shows that the deeper layers offer more resistance; this last character has been established in some cases of carcinomatous epulides.

While they are always fixed upon the gums, epulides do not always have the same *point of origin*. The most of them are implanted on the gums themselves, in the tissue of which they are formed, either preserving the aspect of the tissue, or giving rise to fungosities or hard and embossed tumors. Others evidently spring from the alveolo-dental periosteum ; they originate in the depth of an alveolus, and may present the aspect of the former, or assume the form of fibrous or fibro-cartilaginous tumors. Finally, there are some which originate in the maxillary bones themselves ; these are the gravest of all, for they constantly tend to the destruction and disorganization of the bone on which they are fixed.

The *structure* of the tumors of the gums has hitherto been very little studied ; I have found anatomical characters given in only four reported cases, so that in spite of the microscopic labors of M. Robin, this part of their history is still a desideratum. Finally, considering all the data possessed by science, and reviewing the clinical study I have undertaken in the second part of this work, I consider myself authorized to admit the following species :

1. *Simple epulis,* consisting of a hypertrophy or abnormal development of the proper tissue of the gums.

2. *Fungous epulis,* characterized by a production of fungosities or fleshy vegetations, flabby, spongy, shaped like a mushroom, composed of a great number of vessels and of fibro-plastic tissue.

3. *Vascular* or *erectile epulis,* "the organization of which appears to be the same as that of erectile tumors."

4. *Fibrous, fibro-plastic,* or *fibro-cartilaginous epulis,* composed of a white, compact, incompressible tissue, not capable of being crushed, affording no cancerous juice, but little vascular, with resisting fibres or lamellæ intersecting one another in all directions. It is composed chiefly of fibrous and fibro-plastic elements.

5. *Bony epulis.*—In the single case of this variety with which we are acquainted, the tumor was composed interiorly of bony tissue, with large meshes. Other fibrous or carcinomatous tumors, contain nuclei or concretions of bone, which seems to establish a sort of relation among the different species of epulis.

6. *Carcinomatous epulis.*—This species originates in the bony tissue, which it destroys, and for which it substitutes itself, we have found it formed of a dense, cartilaginiform tissue, resembling lardaceous cancer, and bony in several points, especially towards the central part. These bony concretions are met with only in the free portion of the tumor: that which is implanted in the bone is composed of a soft tissue which stimulates its absorption and leads to the formation of little spiculæ. This has imposed upon certain surgeons by leading them to suppose that the disease depended upon a caries or necrosis of the maxillary bones. It is to these tumors that the micrographical characters described by Mr. Robin belong.

The *symptoms* to which the tissues of the gums give rise, are common to all the species or peculiar to some of them. Their commencement is generally not perceived, when it has not been preceded by a fluxion, an abscess or a dental caries. Nevertheless, acute, severe, lancinating pains, without appreciable lesion of the parts, have sometimes announced their occurrence. When they are still small, they cause little inconvenience; but as they increase, they disturb the functions of neighboring organs. The teeth are at first pushed aside, then loosened and forced from their sockets. Mastication is impaired because the jaws cannot completely close. The tongue being impeded in its motions, speech and deglutition become difficult and painful. When the tumor originates in the inner gums, it may be prolonged upon the pharynx and offer an obstacle to respiration. These inconveniences are less decided when the epulis occupies the external aspect of the gums; but then the teeth and the lips are deformed, the lineaments of the face are altered, the lips pushed forward, the half-open mouth allowing a viscid saliva to escape.

At this stage of the disease, the tumor often ulcerates, is covered with phlyctænæ, and secretes a sanious or puriform matter of an extremely fetid odor. Irritations of all sorts, or simply the motion of eating or speaking occasion a more or less abundant bloody discharge. The pains, usually absent or very slight in fungous and fibrous epulis, are acute, lancinating, increasing at night, in carcinomatous epulis, of which they are one of the essential characters. Engorgement of the submaxillary glands often occurs in this last variety.

The *progress* of epulides is ordinarily slow. They may remain stationary for months and years, and then assume a more rapid development. Some have lasted three, five, ten, and even thirty years. Fungous and fibrous tumors remain longest without serious symptoms. The vascular and carcinomatous are more rapid in their growth, and tend to bring about the destruction of the bones on which they rest; their mean *duration* being from one to two years, though it may be still shorter.

It has been asserted that the epulides which are symptomatic of the caries of one or two teeth, sometimes disappear spontaneously after the extraction of these teeth. This may be true, if the tumor consists only of fungosities of small volume, and even then it requires proof, but it cannot be so with vascular, fibrous, bony or carcinomatous tumors, in a word, with all those in which there are products of new formation. These tumors never retrograde; on the contrary, they always tend to increase on the exterior and on the place of implantation. If art does not intervene to terminate them, they may even occasion death by inanition, by asphyxia, by gradual exhaustion, or by true cachexy.

The *diagnosis* of epulis is drawn from the different circumstances which have preceded, accompanied and followed its development. It cannot be confounded with the swelling of the gums produced by scurvy or the action of mercury. It is distinguished from phlegmon and abscess of the gums, or *parulis,* by the pain, heat, redness and tumefaction, soon followed by an abscess which bursts, giving exit to a greater or less quantity of pus. The other diseases, with which it may be confounded, are exostoses, cysts, fibrous and cancerous tumors of the maxillary bones.

Exostoses of the maxillæ are rare and do not occupy the border of the alveoli; besides they are remarkably hard, have a large base, and make one substance with the bone. Fibrous or carcinomatous tumors of the maxillary sinus sometimes protrude into the mouth through one or more sockets of the teeth; but then there is always a characteristic deformity of the face which indicates the origin of the disease. Fibrous tumors and cancers of the lower jaw begin in the base of this bone; while epulides commence on the surface of the gum or the alveolar border. In maxillary tumors, it is quite late in the progress of the disease that the alveoli are elevated and the teeth raised and loosened; while these phenomena occur at the beginning of epulis.

The *prognosis* of these tumors varies according to the causes which produce them, the nature of the morbid products which compose them, and their point of origin. Those which are evidently induced and kept up by caries of a tooth or of a portion of alveolus, yield readily to art, and do not return when their cause is removed, or they themselves taken away. Simple fungous and fibrous epulides are not serious except from the local disturbances which they induce; their extirpation, properly practiced, is not followed by regermination. Carcinomatous epulides, rising from the jaw bones, on which they are planted, have, in consequence of these circumstances, a certain malignity which may bring about a recurrence after the operation, and even cause death. The same is true of vascular erectile tumors, when they are large and reach the bony tissue.

If, as has been seen, authors disagree as to the nature of the tumors of the gums, it is proper to say that this is not the case as to their *treatment*. The indications are too evident to escape the notice of any. It is necessary, first, to destroy the causes of the disease, if they exist; secondly, to remove the tumor; thirdly, to produce, in the parts from which it springs, a modification which prevents its recurrence.

The extraction of carious or very loose teeth is necessary in all cases of tumors of the gums; the presence of these osteoids, now become really foreign bodies, would be an obstacle to the cure, and might induce a recurrence of the tumor after the operation. Besides, the origin of the epulides being possibly the alveoli, it is necessary to lay them bare in order to cauterize them.

The removal of the tumors may be effected in several ways. Simple tumors, or those which are symptomatic of caries of one or more teeth, if small, may be excised with scissors or a curved bistoury, and then cauterized with nitrate of silver.

The ligature may be applied to pediculated tumors, in timid persons; but excision or evulsion is generally preferable. This method is only really indicated in pedicled erectile tumors, the excision of which might give rise to a hemorrhage; still, the other methods appear to me much surer.

Certain hard, fibrous, and not very vascular tumors, inserted into the gums by a narrow base, may easily be torn out with the fingers or Museux's forceps. "In an officer who came to consult us at Val-de-Grace," says M. Begin, "an epulis of the size of a pigeon's egg, springing from the inside of the right ramus of the lower jaw, pushed aside the tongue, and interfered with its movements. In examining the tumor, I found it supported by a pedicle so narrow and offering so little resistance, that with my finger passed beneath it in form of a hook, I pulled it away without effort, and removed it from the mouth. After a slight hemorrhage the patient went away and experienced no return of his disease."

Excision with the bistoury is the sole process which ought to be used in voluminous tumors with a large base and deeply implanted. We must then carefully remove all the roots of the disease, cutting through the sound parts and exposing the bone, whenever we have reason to believe that the disease extends so far.

Some surgeons, under the erroneous idea that tumors of the gums always spring from the salient part of the alveolar border, have adopted a general absolute rule, always to carry away to a considerable extent the portion of bone which supports them. This plan is adopted by M. Velpeau, who has even contrived a cutting forceps, of a concave form, intended to remove, at a single clip, the tumor and the bony portions on which it is supposed to be fixed. Several members of the Society of Chirurgery, among them Messrs. Verneuil, Gosselin, Forget and Lenoir, share this opinion, and say they have put it in practice with success. I readily admit that so heroic a remedy generally effects a radical cure; but, before employing it, we ought to be sure that it is necessary. The facts which we have accumulated show that if, in some cases, the epulides take root in the alveoli and the maxillary bones, they oftener arise exclusively from the gums or the alveolo-dental periosteum. Why then cut away bony parts which may be advantageously preserved? Besides, we must remark, that in following the process of M. Velpeau, we act by chance: for this surgeon, like the others I have cited, does not appear to have paid special attention to the determination of the clinical characters of the tumors which really originate from the bone. I therefore reject this plan, as a general method of operating on tumors of the gums, reserving its employment only for those cases in which we are sure that the epulis proceeds from the bone.

Excision, or ligature, according to circumstances, may suffice to bring about a cure in those epulides issuing from the proper tissue of the gums, without alteration of the alveolo-dental periosteum of the bones. But, as we cannot be absolutely certain of this, prudence requires the cauterization of the surface whenever they issue, to prevent their recurrence. Caustics applied in the mouth would be liable to too many inconveniences, to allow us to employ them; we must therefore, under these circumstances, have recourse to red hot iron. This is particularly indispensable when we are dealing with epulides complicated by caries of the maxillæ or by carcinomatous tumors. We must then, with a strong bistoury or with a gouge, remove and destroy all the altered bony parts, in order to apply the caustic to the utmost limits of the disease. If the epulis proceeds from an alveolus, we must introduce into its cavity a caustic in a tube, in order not to leave untouched any of the diseased parts. Vascular and erectile tumors also demand the energetic employment of red hot iron, not only to destroy all the altered bony tissue and prevent recurrence, but also to arrest hemorrhage, which might be serious, under such circumstances.

Vascular or carcinomatous tumors, too long neglected or ineffectually treated, may assume such forms of development as will render inapplicable or insufficient the various operations we have just considered. Then the partial or total resection of the maxillæ may become indispensable. It must be unhesitatingly resorted to, if this grave operation is the only chance for safety which remains to the patient.

The treatment after operation is very simple: it consists of rest, a light diet, frequent use of gargles, at first emollient and afterwards detersive. The cicatrization of the wound ought to be attentively watched, in order to keep down the vegetations which may form, and to apply anew the actual cautery if there is any tendency to recurrence of the tumor.

